# Social innovation in diagnostics: three case studies

**DOI:** 10.1186/s40249-020-0633-6

**Published:** 2020-02-19

**Authors:** Megan L. Srinivas, Eileen J. Yang, Priyanka Shrestha, Dan Wu, Rosanna W. Peeling, Joseph D. Tucker

**Affiliations:** 1 Institute for Global Health & Infectious Diseases, University of North Carolina at Chapel Hill, Chapel Hill, NC USA; 2 International Diagnostics Centre, Department of Clinical Research, Faculty of Infectious and Tropical Diseases, London School of Hygiene and Tropical Medicine, London, UK; 3 Special Programme for Research & Training in Tropical Diseases (TDR), World Health Organization, Geneva, Switzerland

**Keywords:** Social innovation, Diagnostics, Malaria, HPV cervical testing, Crowdsourcing, HIV testing

## Abstract

**Background:**

Diagnostics are essential for identifying and controlling diseases. However, limited access to diagnostics hinders public health efforts in many settings. Social innovation may provide a framework for expanding access to diagnostics in the global south. Here social innovation is defined as implementing a known public health tool via a novel, community-driven technique.

**Main Body:**

In this article, we discuss three diverse cases that show the potential for using social innovation in diagnostics. The cases chosen for inclusion here demonstrate the importance of social innovation in diagnostics across different geographic, cultural, and health system contexts. They include malaria testing via schools in Malawi, cervical human papillomavirus (HPV) sample self-collection in Peru, and crowdsourcing human immunodeficiency virus (HIV) testing in China. For each case, we present the public health problem and the impact of using social innovation to increase accessibility of diagnostics. We discuss implications of each diagnostic approach and the importance of social innovation in creating these potential solutions. We argue that social innovation is useful in improving the delivery of essential diagnostic tools in low- and middle-income countries.

**Conclusions:**

Interventions in Malawi, Peru, and China suggest social innovation increases uptake of diagnostics. The same tools and principles utilized in these cases can be adapted for use in other contexts. Such diagnostic innovations may help improve identification of and linkage to care for many diseases. The approach presents a unique opportunity to better address public health issues and increase accessibility in LMIC health systems.

## Background

Diagnostics serve essential functions in health systems, enabling epidemic response, health surveillance, and screening programs. They are also critical for achieving universal health coverage and the United Nations’ Sustainable Development Goal 3: “Ensure healthy lives and promote well-being for all at all ages.” [1] Here we define diagnostics as any equipment, method, or system used for determining a medical diagnosis [2, 3]. One method to enhance the role of diagnostics in health systems is social innovation: implementation of a known public health tool via a novel, community-driven technique [4]. These processes can manifest as an original product, role or behavioral practice, market mechanism, policy, or paradigm shift [5]. Social innovation provides a new lens in addressing health system challenges by engaging communities from the outset in planning, implementing, and institutionalizing an intervention.

Applying social innovation to diagnostic measures can improve healthcare accessibility. For instance, one group of researchers used social innovation to overcome obstacles in tuberculosis (TB) treatment in a project focused on decentralizing TB diagnosis and care for low-income populations in India and Cambodia [6]. Their initiative used local providers to create multiple small Directly-Observed Treatment, Short-course (DOTS) centers at community sites in close proximity to patients’ homes [6]. Additionally, they implemented a text-based digital alert system to create a more timely communication of sputum results to the local providers [6]. These innovations prove to be cost-effective, sustainable, increase access to diagnostics, and strengthen health systems [6].

Despite the growing role of social innovation in diagnostics, few studies have examined this critical interface [5]. Our purpose here is to describe three social innovation cases from different geographic regions and examine each intervention’s respective public health problems, goals, approaches, outcomes, and implications.

## Main text

**Fig. 1:**
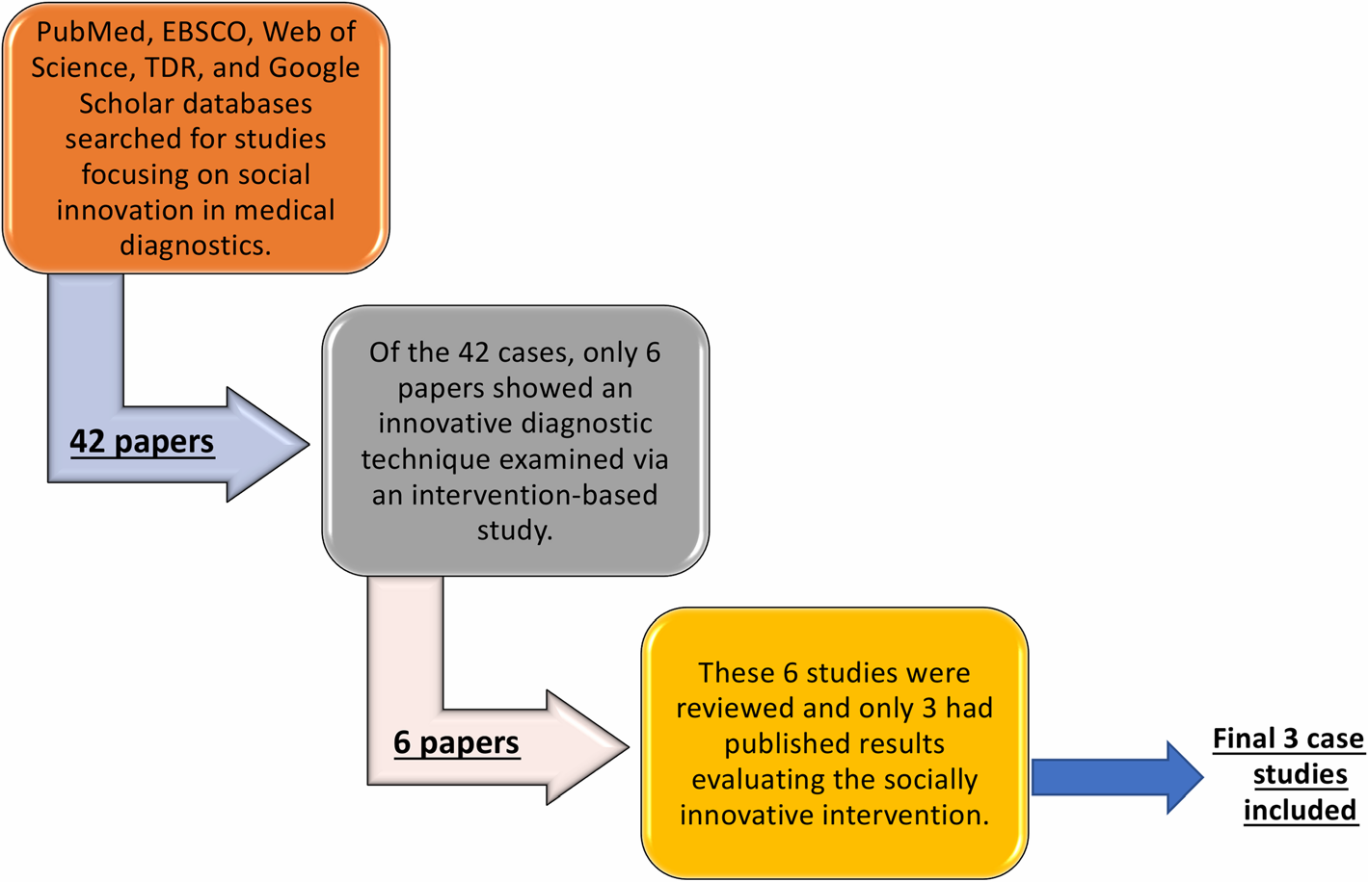
The diagram of cases selection. TDR: Special Programme for Research and Training in Tropical Diseases

We searched for studies focusing on social innovation related to diagnostics. We used PubMed, EBSCO, Web of Science, and Google Scholar databases as well as cases and reports compiled by the World Health Organization’s Special Programme for Research and Training in Tropical Diseases (TDR)/Social Innovation in Health Initiative [7]. We selected cases based on the total number of existing references, availability of data, and relevance to diagnostics. Three final cases were selected based on cumulative evidence related to the profiled social innovations (see also Fig. 1).

**Fig. 2:**
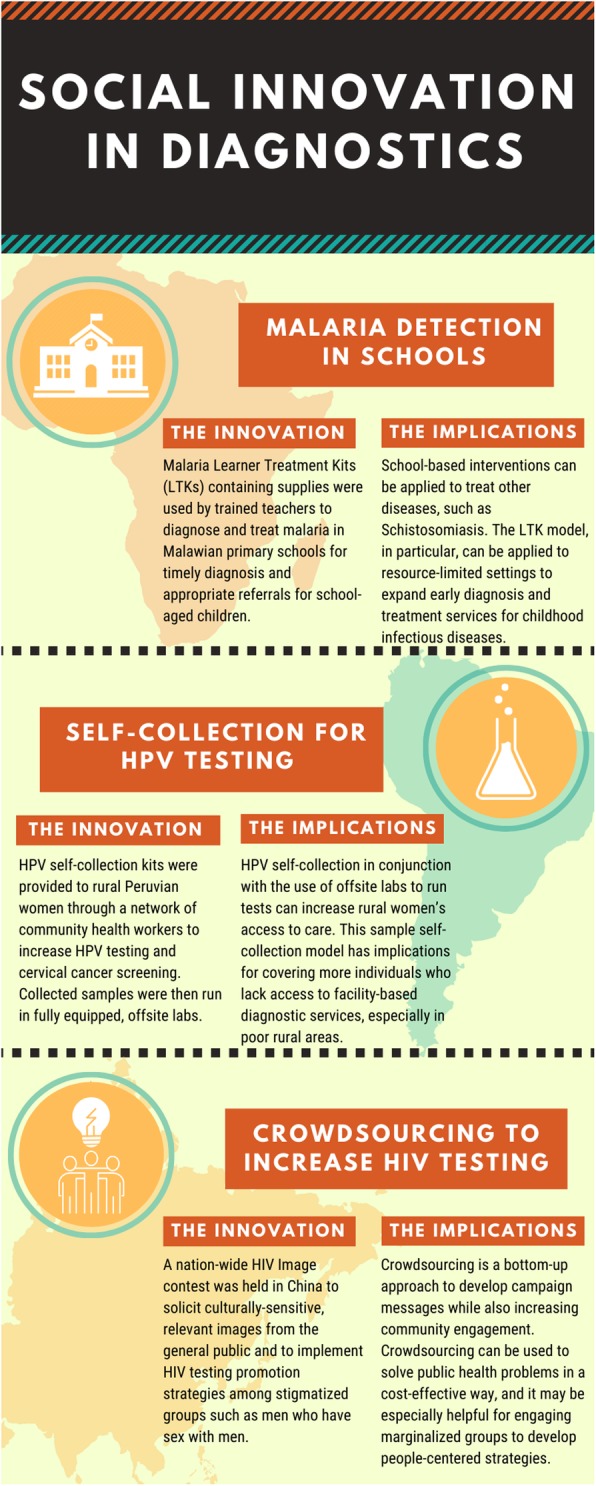
Social innovation in diagnostics for malaria, HPV and HIV. HPV: Cervical human papillomavirus; HIV: Human immunodeficiency virus

Of a total of 42 cases, the three that were selected to highlight in greater detail studied malaria detection in schools, self-collection for cervical human papillomavirus (HPV) screening, and crowdsourcing for human immunodeficiency virus (HIV) testing. These are described below (see also Fig. 2).

### Malaria detection in schools

Sub-Saharan Africa has one of the highest malarial disease burdens in the world [8]. Like many nations in this region, Malawi has implemented preventative measures to reduce occurrence of new malaria infections. Despite these efforts, the malaria incidence and mortality rates in children have increased in the past decade, especially amongst school-age children who are not typically targeted in malaria scale-up programs. One study estimated that 60% of school-age children were infected with malaria, but that most remain undiagnosed and, thus, untreated [9]. Since 2008, the World Health Organization (WHO) has recommended rapid malaria testing prior to initiation of treatment [10], but accessibility to these tests in rural areas is a challenge. Prior studies have attempted to provide community health workers (CHWs) and private drug shop owners with rapid diagnostic tests (RDTs) to broaden access to diagnostics [11, 12]. These efforts were effective in increasing detection and treatment of malaria in the general population, but did not adequately address the gap in diagnostics for school-age children. With malaria causing 50% of all deaths in Africa’s school-age children [13], initiatives are needed to control the disease burden in this age group [14]. While access to health facilities is limited in rural Malawi, there is a high rate of enrollment in primary schools, providing an opportunity for public health interventions [15].

The Learner Treatment Kit (LTK), funded by Save the Children and implemented in conjunction with the Malawi Ministries of Health and Education, the London School of Hygiene and Tropical Medicine, and the University of Malawi, demonstrates a social innovation in malaria diagnostics and treatment [16]. It uses schools as an entry point for care delivery, which both increases healthcare accessibility and incentivizes school attendance. Additionally, it empowers trusted teachers to be care providers.

LTKs are first aid kits that include malaria RDTs [6, 16]. The project made LTKs accessible to primary school children (ages 4–18 years) during normal school hours. Between November 2013 and April 2015, 58 schools in rural Malawi participated in the study. Of these, 29 were assigned to the intervention arm of a pragmatic observational study. At least two teachers per intervention school underwent a 7-day training to become LTK providers for their students. Teachers learned how to diagnose (using RDTs) and treat malaria and other minor illnesses, and completed a three-day mentorship at a local health center. Following training completion, students and parents were notified that selected teachers were available at their schools to enhance health and well-being. Students were instructed to approach any of these trained teachers during the school day if they felt unwell. If appropriate, the teacher administered a RDT and, pending results, started antimalarials. If a student’s presentation appeared beyond the teacher’s level of training, the teacher contacted the student’s parent/guardian and referred him/her to a local health center for care along with documentation describing the reason for referral. Teachers were evaluated at different points during the intervention to ensure continued competency. At each evaluation, the trained teachers demonstrated sufficient knowledge to appropriately diagnose and treat simple malaria cases [17].

The LTK project’s efficacy was initially evaluated via two methods: 1) eight focus groups of school-age children, parents/guardians, and teachers, and, 2) 20 in-depth interviews with key stakeholders at the school, district, and national levels [16]. This perception analysis demonstrated the LTK project’s success in increasing accessibility to malaria diagnostics and treatment [16]. Students reported that they sought care more frequently when feeling unwell [16]. The intervention was associated with a reduction in school absences due to health issues and a reduction in students dropping out of school or repeating grades [17]. Both parents and students reported that they trusted the teachers selected to provide malaria services [16]. Additionally, health care workers at local and regional clinics stated that the LTK program helped decrease unnecessary visits to their already overburdened clinics [16].

The innovation behind the LTK has implications for malaria detection and treatment. For many diseases, diagnostic tools already exist that enable accurate and timely identification. However, while these tests may be available, they are not always accessible where needed in resource-poor settings [17]. The LTK attempts to address this by enabling increased access to the diagnostics for malaria in rural Malawi [17]. School-based interventions have been successfully used to treat schistosomiasis and other soil-based helminths throughout the world [18–20]. The LTK innovation goes a step further and demonstrates the ability to implement diagnostics as well as treatment through a school-based program. Additionally, while previous antimalarial strategies have focused on pregnant women and children below five years of age [21], this innovation addresses the high burden of malaria amongst school-age children, drawing attention to this important and frequently overlooked subgroup. The innovation also demonstrates the ability to train community members who are not typically involved in healthcare, and shows that their inclusion enables a vital extension of services in rural areas where access to care is limited.

### Self-collection for cervical human papillomavirus (HPV) screening

Medical technology advancements are improving the ability to prevent cervical cancer via early detection and management of the HPV [22, 23]. Specifically, the introduction of cervical HPV testing has decreased the incidence of cervical cancer in areas where it is used. However, many LMICs continue to have high mortality from cervical cancer because of poor HPV screening availability [24, 25]. Even where medical exams are available, many women are not properly referred for HPV screening. Additionally, the delivery and transport of sample collection kits for HPV testing is often difficult in rural and impoverished areas, decreasing access to screening in many settings.

Due to many of these issues, HPV screening in Peru remains low, causing the country to have a significantly higher incidence of cervical cancer as compared to its South American counterparts (34.5 cases per 100 000 women versus 24.1 cases per 100 000 women) [24]. The Peruvian Cervical Cancer Screening Study introduced a novel solution to this problem: have community health workers in resource-poor areas distribute cervical sample self-collection kits to women, thereby avoiding the need for medical examinations and HPV screening referrals.

Two Peruvian sites were chosen for the study, a poor suburban village and a remote village near the Amazon [26]. CHWs provided self-collection HPV testing kits to women who enrolled in the study at each site. Each kit contained a brush for sample collection, a specimen card, and simple illustrated instructions. Women collected the samples at home and returned the specimen cards to the CHWs. The CHWs sent the specimens via mail to a centralized laboratory to undergo HPV testing. Community health workers received all test results and relayed all results to the women. Women who tested positive for HPV were referred to regional health clinics for further evaluation and treatment.

A total of 643 women registered to take part in the study with 632 women (98.3%) returning their samples to the community health workers [26]. Fifty-nine (86.8%) of the 68 women who tested positive for HPV followed through with referral for evaluation and treatment at a regional health center. The majority of women in the study (81.4%) preferred sample self-collection rather than going to a clinic for a traditional examination. Given the ease of distribution and collection, this intervention increased the reach of screening diagnostics for HPV in both indigent and rural populations. Additionally, this innovative approach improved linkage to care for those at a higher risk for cervical cancer.

This innovation demonstrates the power of a bottom-up approach that enables patients to have control over their own health. Such approaches increase patients’ agencies in health services and promote their engagement in other health-related behaviors. Given the initial effectiveness, a second iteration included referring local adolescent women to initiate HPV vaccination series [22, 23]. This innovation is easily adapted for different settings. Uganda [27], Haiti [28], and Argentina [25] have all successfully applied similar self-collection HPV screening strategies.

### Crowdsourcing to increase HIV testing

Globally, men who have sex with men (MSM) are 19 times more likely to have human immunodeficiency virus (HIV) than the rest of the population [29]. In high-income countries, systemic preventative interventions have substantially decreased new HIV infections. However, in LMICs, interest in addressing HIV is often low due to the continued marginalization of key populations, pervasive social stigma surrounding lesbian, gay, bisexual, and transgender (LGBT) communities, and lack of gay-friendly clinical services [30]. Negative social attitudes towards gay men have contributed to low HIV testing rates, limiting the effectiveness of HIV treatment and prevention efforts [31]. In China, traditional top-down approaches to increase HIV testing among key demographics like MSM have been unsuccessful, and HIV infection incidence has increased among MSM in recent years [31]. Thus, promotional interventions that can more effectively reach MSM are needed.

The Social Entrepreneurship to Spur Health (SESH) group applied an innovative crowdsourcing approach to gather promotional materials from the public and increase community awareness of and engagement with HIV testing. Crowdsourcing draws upon the knowledge and creativity of both experts and members of the public to come up with innovative solutions, which are then shared and implemented to benefit the wider community [32]. The SESH team issued an innovation challenge that allowed the community to develop solutions in response to an open call.

The National HIV Testing Image Contest, held in China in 2016, solicited images from the Chinese public that would effectively and creatively promote HIV testing among the Chinese MSM population [31]. The contest was advertised both online through Chinese social media sites – WeChat, Weibo, and QQ – and in-person at events held by community-based organizations in four major Chinese cities. After a six-week open-call period, submissions were screened for eligibility. Eligible submissions were evaluated by a panel of judges that included local MSM, public health researchers, and media experts, with entries scored on the basis of novelty, relevance, feasibility, and elaboration. In total, 431 submissions were received from across the nation. Finalists were awarded cash and material prizes for their work, and the five finalists’ submissions were shared with the public through social media in order to promote HIV testing. The top five images were then used in tandem with two other crowdsourcing contests — a HIV testing story contest and a regional HIV diagnostics delivery designathon — to form a comprehensive HIV testing intervention package [31]. Ultimately, the comprehensive intervention led to an 8.9% absolute increase and 43% relative increase in HIV testing among MSM, which was non-inferior to conventional HIV promotion interventions [31]. The crowdsourced intervention, however, was shown to be particularly effective at increasing HIV self-testing rates over facility-testing rates, with 49% of participants reporting using self-testing services [31]. Additionally, 62% of the cohort was tested for HIV at least once during the study period, and 56% of previously untested MSM in the study received HIV testing [31].

Using a challenge contest to develop promotional HIV testing materials lowered the costs of messages while proving to be as effective as conventional interventions [31]. Crowdsourcing may be a more sustainable, cost-effective method to develop health campaigns. When adapted into a challenge contest-, crowdsourcing allows for community members to be more directly involved in the development and implementation of public health interventions [33]. Challenge contests have the potential to produce innovative and culturally sensitive solutions by drawing on the public’s creativity and knowledge. These solutions can effectively appeal to targeted populations [33]. Crowdsourced interventions can be especially helpful in promoting awareness of stigmatized diseases, as they can reach specific populations while simultaneously changing public perception and increasing community acceptance. Crowdsourcing can also be used to address a variety of health issues. Beyond propagating HIV testing, crowdsourcing has been used to facilitate the development of promotional materials for diseases such as Hepatitis B and C [34].

## Discussion

The three cases examined in this article reveal the potential of social innovations to address equity issues in access to diagnostic testing. Through innovative methods, sustainable and scalable practices (see Table 1 on scalability), and engaging the community to take a more active role in healthcare, these innovations successfully increased the utilization of diagnostics amongst vulnerable populations within LMICs. The Malaria LTK innovation in Malawi increased access to malaria care for school-aged children by recruiting and training teachers to use RDTs and provide antimalarials. The Peruvian HPV self-sample collection study made HPV diagnostics much more accessible to indigent and rural patients through self-collection of cervical samples that were sent to overseas labs for analysis. The National HIV Testing Image Contest held in China successfully solicited culturally appropriate HIV testing promotional materials from the general public through crowdsourcing techniques, ultimately increasing the reach of HIV diagnostics.

**Table 1:** Scalability in social innovation

	Social innovation approach/model	Current scope	Potential for scale up
Malawi - malaria LTK	Utilizing teachers to diagnose/treat malaria in school-age children	This specific study was conducted in Malawi, but similar models targeting malaria and other parasites have been successfully implemented in other countries as well.	Similar interventions could be implemented in schools in malaria endemic regions to improve rates of malaria diagnosis/treatment amongst school-age children.
Peru - HPV cervical sample self collection	Increasing HPV testing rates in rural Peruvian women through facilitating self-collection of cervical samples	This intervention took place in rural and resource-poor suburban communities within Peru. Similar interventions have been used in Uganda, Haiti, and Argentina.	Self-collection sampling techniques could be relevant in many areas where there is poor uptake of HPV screening and strong networks of local women to run and support the program.
China - crowdsourcing for HIV testing	Creating more culturally sensitive and impactful materials to promote HIV testing among MSM through an online crowdsourcing contest	This crowdsourcing contest was held in China, but crowdsourcing challenge contests in various forms have been successfully conducted in many global contexts (HIV testing RCT trial, protein folding/genome hackathons, etc.).	Crowdsourcing challenge contests could be useful in soliciting promotional materials for a variety of stigmatized diseases, such as hepatitis B/C and syphilis.

*HPV* Human papillomavirus, *HIV* Human immunodeficiency virus, *LTK* Learner Treatment Kit

One major problem in the design of social innovations is sustainability. Health-focused interventions are often implemented in communities for only a short period of time, ending when funding runs out or the research to which they are tethered is completed. Although these interventions may temporarily improve a community’s health status, these improvements do not last without continued support, training, and resources. We need to focus on creating sustainable social innovations with a lasting impact in the communities in which they are implemented. In order to do so, a few major factors need to be considered: intervention cost, targeted community’s involvement/training/support system, and national policy/infrastructure. In the Peruvian HPV self-collection case study, samples were sent to centrally located labs to be processed, avoiding the need for regional medical laboratory facilities near targeted communities. Moreover, this reduced the local operational costs of diagnosis, making it a practical and fiscally-effective solution. The HIV crowdsourcing contest in China was also a cost-conscious, sustainable solution. By involving the community in creating promotional materials, the crowdsourcing contest reduced the usual developmental costs of materials and increased community investment in a major public health concern. The Malawi malaria LTK case study used community engagement as well to ensure sustainability; by directly training school teachers to be the providers for malaria diagnostics and treatment, the intervention was able to reach a previously overlooked population and maintain their access to care long-term.

This analysis does have limitations. Most importantly, there is a lack of available follow-up data on the members of communities targeted by these social innovations. Downstream data on the frequency and efficacy of treatments provided as a result of these novel approaches in diagnostics would enable a better understanding of the true impact social innovation can have in healthcare delivery. Another major limitation is the lack of a standardized methodology to evaluate social innovations. As a field, social innovation is relatively new, and, thus, there aren’t standardized metrics for evaluating these interventions, making it difficult to directly compare different innovations.

## Conclusions

These three cases demonstrate how social innovation can enhance diagnostic access among vulnerable groups. Social innovations may be useful to make diagnostics more user-centered and feasible in low- and middle-income countries. These cases have implications for new programs and novel research on diagnostics, and demonstrate the nature of and processes that can address inequity and promote the sustainability of social innovations. As can be seen in the approaches here, social innovation in diagnostics is needed to enhance future health coverage and build more resilient health systems.

## Data Availability

Data sharing is not applicable to this article as no datasets were generated or analysed during the current study.

## Abbreviations

CHW: Community health worker
DOTS: Directly-Observed Treatment, Short-course
HIV: Human immunodeficiency virus
HPV: Human papillomavirus
LGBT: Lesbian, gay, bisexual, and transgender
LMIC: Low- and middle-income country
LTK: Learner Treatment Kit
MSM: Men who have sex with men
RDT: Rapid diagnostic test
SESH: Social Entrepreneurship to Spur Health
TB: Tuberculosis
TDR: Special Programme for Research and Training in Tropical Diseases
WHO: World Health Organization
